# The role of acupuncture as a complementary therapy for sepsis: a narrative review of the evidence and future trial directions

**DOI:** 10.3389/fmed.2026.1823604

**Published:** 2026-05-19

**Authors:** Li-Yuan Zhuo, Xin Bao, Ming-Xi Lai, M. Adeel Alam Shah, Xiao-Feng Chen, Shan Jiang, Zhen-Yang Wei, Rong-Lin Chen, Shan-Qiang Zhang

**Affiliations:** 1 Department of Critical Care Medicine, Longgang Central Hospital, Shantou University Medical College, Shenzhen, China; 2 Department of Pathology, Longgang Central Hospital, Shantou University Medical College, Shenzhen, China; 3 Department of Internal Medicine, Baoan Maternal and Child Health Hospital, Shenzhen, China; 4 Department of Anatomy, Shantou University Medical College, Shantou, China; 5 Department of Burn Unit, Longgang Central Hospital, Shantou University Medical College, Shenzhen, China

**Keywords:** acupuncture, clinical trial, meridian, sepsis, traditional Chinese medicine

## Abstract

Sepsis is a life-threatening condition caused by a dysregulated host response to infection. Current interventions are insufficient to halt rising morbidity and mortality, prompting interest in complementary therapies including acupuncture. This narrative review systematically searched clinical studies published from 2010 to 2026 evaluating acupuncture as a complementary therapy for sepsis. Seventeen studies met inclusion criteria. We analyzed study design, participant characteristics, intervention modalities, acupoint selection, outcome measures, and safety, and explored connections between traditional Chinese medicine and modern biomedical mechanisms. The available evidence is insufficient to recommend routine acupuncture use for sepsis. High-quality trials are needed, adhering to Standard for Reporting Interventions in Clinical Trials of Acupuncture (STRICTA) and Consolidated Standards of Reporting Trials (CONSORT) guidelines, and using the Oxford Center for Evidence-Based Medicine Level of Evidence Scale to screen optimal acupoint combinations. This review synthesizes existing clinical evidence and provides a rigorous reference for designing future trials.

## 1 Introduction

Sepsis is a life-threatening organ dysfunction syndrome caused by a dysregulated host response to infection (1). Although well-established risk factors have been identified, including age (infants and elderly), impaired immunity, diabetes, cirrhosis, long-term intensive care, trauma, invasive treatment, and chronic glucocorticoid use, the underlying etiology remains incompletely understood (2–6). As a result, sepsis incidence remains high worldwide, with 15 per 1,000 in-patients requiring costly medical care (7, 8). Moreover, progression to severe sepsis and septic shock involves organ dysfunction and circulatory failure, dramatically increasing mortality risk. In fact, global estimates from 2020 recorded 48.9 million new sepsis cases and 11 million sepsis-related deaths, accounting for nearly one in five deaths worldwide (9). Within intensive care units (ICUs), 20%–30% of patients develop sepsis, and 25%–40% of these eventually die (10). Compounding this burden, survivors often face long-term cognitive impairment and functional disability, imposing substantial medical and social costs (11, 12). Therefore, refining current therapeutic strategies is crucial to improve sepsis outcomes.

Understanding the mechanisms of sepsis is essential for developing better treatments. Although these mechanisms remain incompletely clarified, evidence implicates complex regulatory networks involving inflammation imbalance, immune dysfunction, mitochondrial damage, coagulation disorders, neuroendocrine-immune abnormalities, endoplasmic reticulum stress, and autophagy (13, 14). In response to these pathways, modern medical strategies target hemodynamic, infection, host response, and energy supply (15). However, these approaches cannot fully address acute organ dysfunction or sequelae such as neurocognitive impairment, mental disorders, and ICU-acquired weakness (ICUAW). To illustrate, antibiotics such as ceftazidime-avibactam, vancomycin, and posaconazole cannot eliminate inflammatory storms and may promote drug-resistant microorganisms with long-term use (16). Similarly, fluid resuscitation, while effective for correcting hypovolemia, can worsen gas exchange, renal function, wound healing, and neurocognition when administered excessively (17). Likewise, vasopressors may cause exaggerated vasoconstriction, reducing organ and tissue perfusion (15). Consequently, the U.S. Food and Drug Administration (FDA) has approved no drug specifically for sepsis to date (14, 15, 18). Given these pharmacological limitations, patients increasingly turn to complementary and alternative medicine (CAM) to enhance therapeutic effects.

Among the available CAM therapies, each has distinct advantages and drawbacks. Probiotics can prevent infections but have limited target-organ action (19–22). Early mobilization improves functional outcomes but requires multidisciplinary teamwork, which is often constrained by limited resources (23, 24). Psychological interventions need sustained cooperation from families and staff, posing implementation challenges (25). Herbal remedies such as *Danggui* and *Danshen* exhibit anti-inflammatory effects, yet they require further clinical translation before widespread use (26, 27). In contrast to these approaches, acupuncture offers several practical advantages, including convenience, safety, low cost, rapid results, and strong controllability.

Acupuncture is a key component of traditional Chinese medicine (TCM), guided by meridian theory and syndrome differentiation (28, 29). During a typical session, the practitioner inserts needles at specific acupoints, manipulates them using twirling, lifting, and thrusting techniques, and uses the patient’s “*De-qi*” sensation as the standard for judging treatment response (30). Although practiced in China for millennia, acupuncture is now used worldwide. Notably, the FDA approved acupuncture needles as Class III medical devices in 1979 (31); currently, 22 states license acupuncturists through medical boards, and 48 states have statutes regulating licensure and training (32). Despite these regulatory achievements, acupuncture has not been recognized as an authoritative CAM recommendation for sepsis. This gap in recognition stems largely from its unique theoretical basis and weak evidential foundation, and it serves as the main motivation for the present review.

Modern medicine and TCM approach disease from fundamentally different perspectives. Modern medicine focuses on molecular mechanisms and clinical manifestations, which can quickly address symptoms but often fail to identify or treat underlying causes (33). TCM, in contrast, emphasizes holistic concepts, *Yin-Yang* balance, and syndrome differentiation, aligning closely with individualized therapy (34). Interestingly, sepsis symptoms correspond to the TCM category of “febrile disease” (温病), first described in the classic Treatise on Febrile Diseases (35, 36). Based on syndrome differentiation, sepsis comprises three types: toxic-heat syndrome (毒热证), blood-stasis syndrome (血瘀证), and acute deficiency syndrome (急性虚证). These are treated respectively with heat-clearing and detoxifying (清热解毒), blood-quickening and stasis-dispelling (活血化瘀), and energy consolidating (扶正固本) strategies (36, 37). To implement these strategies, acupuncturists select acupoints on the scalp, ear, tongue, trunk, and limbs, often requiring multiple sessions (38–40). Furthermore, technological advances have produced laser acupuncture (LA), electroacupuncture (EA), and transcutaneous electrical acupoint stimulation (TEAS), all of which have been applied clinically (41, 42). However, as TCM transitions from experience-based to evidence-based medicine, substantial heterogeneity in study design has emerged across clinical trials (43, 44). Thus, thorough analysis of prior studies is essential for designing future high-quality research.

Previous reviews of acupuncture for sepsis have not deeply interpreted relevant TCM knowledge, particularly the functions of specific acupoints and their potential connections to modern molecular mechanisms (45–47). To address this gap, we review clinical studies on acupuncture for sepsis published over the past 15 years. These include randomized controlled trials, double-blinded trials, prospective single-blinded controlled studies, and retrospective studies of manual acupuncture (MA), EA, LA, and TEAS as complementary therapies. Accordingly, this narrative review aims to: (1) systematically identify and describe clinical studies of acupuncture for sepsis published from 2010 to 2026; (2) critically analyze study design elements, including participants, interventions, acupoint selection, outcomes, and safety; (3) synthesize patterns and inconsistencies in the evidence; and (4) provide specific, actionable recommendations for future high-quality trials.

## 2 Methods

Due to the limited number of clinical studies on acupuncture for sepsis over the past 15 years, we did not impose strict quantitative selection criteria. A more restrictive approach would have limited readers’ ability to fully grasp the current state of research. Given the exploratory nature of this review, we employed a comprehensive and inclusive search strategy covering both English and Chinese publications to provide a panoramic overview of the field (22, 44–47).

### 2.1 Search strategy and databases

The search timeframe was set from January 1, 2010, to 2026. Systematic queries were conducted in PubMed/MEDLINE, Embase, and Cochrane CENTRAL for English publications, and China National Knowledge Infrastructure and Wan fang Data for Chinese publications. For Chinese articles, we selected those with English abstracts available in international databases. The search strategy combined terms for diseases and interventions using the following keywords: (“sepsis” OR “septic shock” OR “sepsis-associated encephalopathy” OR “sepsis-induced myopathy” OR “septic gastrointestinal dysfunction”) AND (“acupuncture” OR “electroacupuncture” OR “manual acupuncture” OR “needling” OR “electro-needling” OR “transcutaneous electrical acupoint stimulation” OR “TEAS” OR “laser acupuncture”).

### 2.2 Inclusion and exclusion criteria

We included clinical studies evaluating acupuncture for human sepsis, including randomized controlled trials, non-randomized controlled studies, prospective single-blinded controlled studies, retrospective studies, case series, and observational studies, published from 2010 onward, and written in English or Chinese. We excluded animal experiments, purely basic research, and studies in which acupuncture was used only for symptoms unrelated to sepsis pathophysiology. No strict quantitative restrictions (e.g., sample size cut-offs) were applied. All studies meeting the basic clinical and temporal criteria were retrieved and evaluated in full.

### 2.3 Screening and data extraction

Two authors independently screened titles, abstracts, and full texts, with disagreements resolved by consensus. Extracted data included first author, year, country, sample size, study design, participant characteristics, intervention details (modality, acupoints, frequency, duration), control group, outcome measures, adverse events, and follow-up duration.

### 2.4 Data synthesis and presentation

**FIGURE 1:**
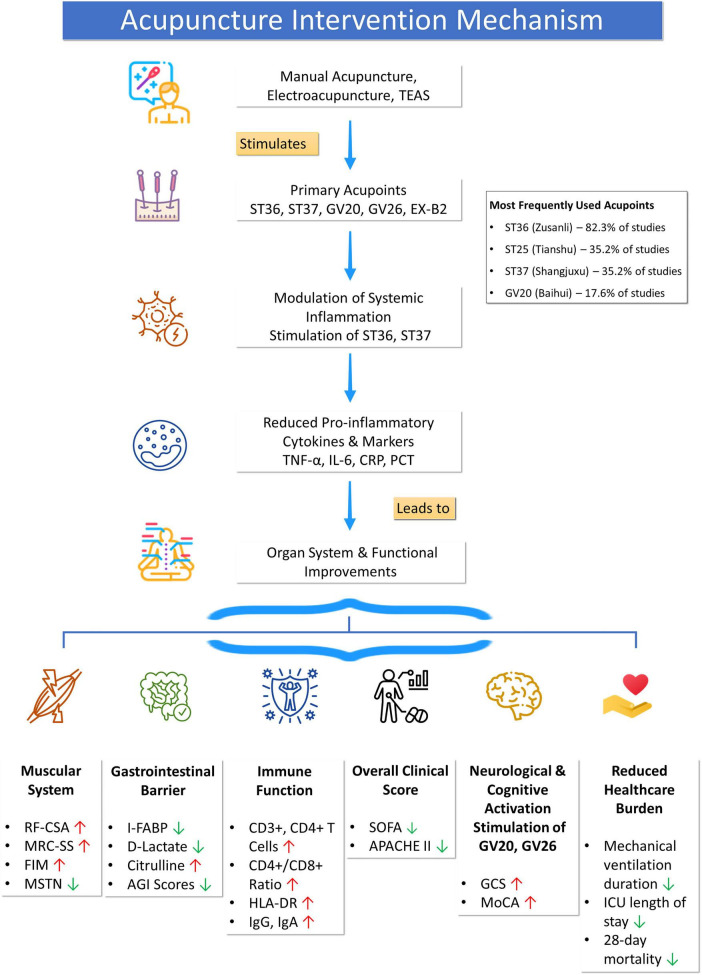
Summarizes the various acupuncture modalities, acupoints, proposed mechanisms, and organ-specific and systemic outcomes investigated in this review.

The key considerations of this review are summarized in Table 1, which provides an overview of the 17 included studies. The acupoints stimulated, along with proposed mechanisms and outcomes, are presented in Figure 1. Due to heterogeneity in study designs and outcome measures, a narrative synthesis approach was adopted rather than a formal meta-analysis (48–50).

**TABLE 1:** The studies on acupuncture in the treatment of sepsis.

Clinical study	Study design	Grouping and number of patients	Therapeutic method	Frequency	Total treatment times	Acupoints		Efficacy evaluation measures	Improvement rate (%)	Adverse effects
						Respective	Frequently used (%)			
Xu et al. (50)	A protocol for an open-label randomized control	CG: 100 EA: 100	WM, WM + EA	2/day	14	ST36, RN4	ST36 (82.3), ST25 (35.2), ST37 (35.2), GV20 (17.6), RN12 (17.6), RN4 (17.6) RN10 (11.8), RN6 (11.8), SP9 (11.8), PC6 (11.8), ST40 (11.8), GB30 (11.8), GV26 (11.8), EX-B2 (11.8), GB34 (11.8), RN23 (11.8)	10-days incidence rate of S-AGI, clinical indicators, plasma concentration of motilin, gastrin and prealbumin, PICS, days on MV, admission time of ICU, mortality 7 d, mortality 28 d	Potentially reduce the occurrence rate of S-AGI to 50%.	Non-reported
Sun et al. (67)	Randomized control	CG: 26 EA: 27	WM, WM + EA	1/day	14	CV4, RN4, RN12, ST25, ST36, ST37, SP9		AGI grading, IAP, intestinal sounds, enteral nutrition pump speed, CRP, PCT, APACHE II, SOFA, mortality 28 d	Non-reported	Non-reported
Liao et al. (68)	Randomized control	CG: 33 MA: 33	WM, WM + MA	1/day	5	Old ten needles: RN13, RN12, RN10, ST25, RN6, PC6, ST36		The bowel sound grading, AGI grading, APACHE II scores, PCT, CRP	Non-reported	Non-reported
Wei et al. (69)	Randomized control	CG: 32 MA: 32	WM, WM + MA	1/day	7	ST36, ST37, RN12, ST25		Changes of bowel sounds, WBC, CRP, PCT, N/L ratio, the feeding time, MV time, admission time of ICU	Non-reported	Non-reported
Li et al. (70)	Randomized control	CG: 29 MA: 28	WM, WM + MA	1/day	7	RN12, RN10, ST25, RN4, RN6, ST36, SP4		APACHE II, abdominal circumference, IAP, intestinal sounds, WBC, CRP	Non-reported	Non-reported
Chen et al. (58)	A single center, propensity-score stratified, assessor-blinded, prospective pragmatic controlled trial with a 1-year follow-up period	CG: 49 MA: 49	WM, WM + MA	2/day	28	ST36, ST40, LI10, GV20, GV24, EX-HN3, TE5, LI4, LI11, LU5, GB34, SP10, ST34, GB30, Allergy Point, Shenmen Point, Thymus Point, ACTH Point		RF_*CSA*_, MRC-SS, FIM™, MSTN, SOFA, APACHE-II scores, MV duration, ICU length of stay, Patient follow-up.	Non-reported	Non-reported
Zheng et al. (59)	Randomized control	CG: 35 EA: 35	WM, WM + EA	2/day	14	GV20, GV26		CRP, IL-6, NSE, MoCA, GCS	WM: 20/35 (57.1%), WM + EA: 31/35 (88.6%)	Non-reported
Liu et al. (60)	A multi-center randomized control	CG: 25 TEAS: 24	WM WM + TEAS	2/day	10	ST25, ST37, ST41, SP8, ST36, CV12, SP15		MI, AGI, duration of EN, hospitalization time, admission time of ICU	Non-reported	Non-reported
Wang et al. (57)	Randomized control	CG: 30; EA: 28	WM WM + EA	2/day	14	GB30, ST32, ST36, GB39, LR 3		MRC, MRS, bilateral quadriceps thickness, gastrocnemius pinnate angle, admission time of ICU, hospitalization time, mortality during hospitalization, mortality 28 d	Non-reported	Discomfort (1 patient)
Li et al. (61)	Randomized control	CG: 20 MA: 20	WM, WM + MA	1/day	10	EX-B2		APACHE-II scores, IAP, intragastric residual volume, borborygmus levels	None-reported	None-reported
Li et al. (55)	Randomized control	CG: 59 MA: 59	WM, WM + MA	1/day	10	EX-B2		WBC, hs-CRP, PCT, enteral nutrition feeding dose, gastrointestinal dysfunction score	None-reported	None-reported
Lin et al. (62)	Randomized control	CG: 32 MA: 32	WM + CMA, WM + Tongdu Tiaoshen MA	1/day	10	GV20, GV26, DU16, DU24, DU14, DU11, RN23, GB20, GB12, PC6, SJ17, ST40, SP9, RN22, RN23, LI11, LI4, BL17, BL15, BL20, SP6, BL18, BL23, ST36		MoCA, IL-6, CRP, Lac	None-reported	None-reported
Meng et al. (65)	Randomized control	CG: 36, EA: 35	WM, WM + EA	2/day	10	ST36, ST37		PCT, TNF-α, I-FABP, D-lactate, Citrulline, TCM quantitative score of intestinal dysfunctions, Days on MV, Length of stay in ICU, 28 d mortality	None-reported	None-reported
Meng et al. (63)	Randomized control	CG: 41; EA: 41	WM WM + EA	2/day	10	ST36, ST37		TNF-a, IL-1b, IAP levels, days on MV, length of stay in ICU, 28 days mortality	None-reported	None-reported
Yang et al. (49)	Randomized control	CG: 29 EA: 29	WM, WM + EA	2/day	14	ST 36, RN 4		None-reported	None-reported	None-reported
Xiao et al. (65)	Randomized control	CG: 30, Thymosin α1: 30, MA: 30	WM, Thymosin α1, WM + MA	1/day	6	ST36, GB34, PC6, RN4		CD3+, CD4+, CD8+, CD4+/CD8+, IgG, IgA, IgM, Length of ICU hospital stay, hospital readmission rate, Mortality 28 d	None-reported	None-reported
Wu et al. (66)	Randomized control	CG: 24, EA: 26	WM, WM + EA	2/day	6	ST36, ST25, ST37, ST39		L/M, D-lactic acid, Time of target treatment, Total effective rate	None-reported	None-reported

MA, manual acupuncture; CG, control group; EA, electroacupuncture; WM, Routine Western medicine; CMA, conventional manual acupuncture; CD3+, percentage of CD3+ lymphocytes; CD4+, percentage of CD4+ T lymphocytes; CD8+, percentage of CD8+ T lymphocytes; CD4+/CD8+, CD4:CD8 ratio; WBC, white blood cells; NE%, percentage of neutrophils; NE, neutrophils; LY%, percentage of lymphocytes; LY, lymphocytes; N/L ratio, ratio of neutrophils to lymphocytes; APACHE-II, acute physiology and chronic health evaluation-II; SOFA, Sequential Organ Failure Assessment; ICU, intensive care unit; RFCSA, rectus femoris cross-sectional area; FIM, Functional Independence Measure; MRC-SS, Medical Research Council Sum-Score; MSTN, myostatin; SIM, sepsis-induced myopathy; CRP, C-reactive protein level; IL-6, Interleukin-6; NSE, Neuron-specific enolase; MoCA, Montreal Cognitive Assessment; GCS, Glasgow Coma Scale; TEAS, transcutaneous electrical acupoint stimulation; MI, motility index; EN, enteral nutrition; AGI, acute gastrointestinal injury; MRC, Medical Research Council; MRS, Modified Rankin Scale; Lac, lactic acid; I-FABP, intestinal fatty acid-binding protein; TNF-α, tumor necrosis factor-α; PCT, procalcitonin; TCM, traditional Chinese medicine; MV, mechanical ventilation; HLA-DR, human leukocyte antigen-DR on monocytes; L/M, lactulose to mannitol excretion ratio; IgG, immunoglobulin G; IgA, immunoglobulin A; IgM, immunoglobulin M; IAP, intra-abdominal pressure; S-AGI, septic acute gastrointestinal injury; PICS, persistent inflammation, immunosuppression, and catabolism syndrome; ST36, ST36 acupuncture point; ST40, ST40 acupuncture point; LI10, LI10 acupuncture point; GV20, GV20 acupuncture point; GV24, GV24 acupuncture point; EX-HN3, EX-HN3 acupuncture point; TE5, TE5 acupuncture point; LI4, LI4 acupuncture point; LI11, LI11 acupuncture point; LU5, LU5 acupuncture point; GB34, GB34 acupuncture point; SP10, SP10 acupuncture point; ST34, ST34 acupuncture point; GB30, GB30 acupuncture point; Allergy Point, Allergy point; Shenmen Point, Shenmen point; Thymus Point, Thymus point; ACTH Point, ACTH point.

### 2.5 Quality assessment

We assessed reporting quality using STRICTA and CONSORT checklists (51–54) and the Oxford Center for Evidence-Based Medicine Scale. Formal scoring was not performed due to the narrative nature of this review.

## 3 Results

### 3.1 Study participants

Only one of the 17 included trials enrolled more than 100 participants; sample sizes ranged from 30 to 120 (median = 60) (55). None reported *a priori* sample size calculations based on effect size (56). Age reporting was inconsistent: Wang et al. (57) set no upper age limit (mean age 43 years), Chen et al. (58) included patients up to 85 years, and another Wang et al. (57) also set no maximum age. Moreover, no study consistently handled age as a covariate or stratification factor. Most trials also lacked strict inclusion criteria for specific organ dysfunctions such as sepsis-associated encephalopathy (SAE), ICUAW, or gastrointestinal failure (49, 58–60).

### 3.2 Group setting

All 17 studies reported random assignment, and most described single- or double-blinding (49, 50, 55, 57–70). Small-sample trials typically used simple randomization. Every study included a control group (usual care or sham intervention). However, Lin et al. (62) used acupuncture in both experimental and control groups (differing acupoint combinations), a design suitable for studying acupoint specificity in SAE but incapable of proving additional benefit over standard care alone. Notably, none of the studies incorporated a true sham acupuncture control (placebo needles, superficial needling, or non-acupoint stimulation). Only one trial, by Xiao et al. (65), included an active non-acupuncture comparator (thymosin-α1) alongside standard care.

### 3.3 Intervention modalities

All experimental groups received acupuncture plus standard modern medical therapy (antibiotics, fluid resuscitation, vasopressors) (15–17, 71, 72). No study explicitly reported whether patients received other CAM therapies (e.g., probiotics, herbal remedies) (19, 21, 22, 73, 74). Among the 17 studies, 7 used EA and 10 used MA; none directly compared EA versus MA within the same trial. Treatment regimens varied substantially: Wu et al. (66) used two 60-min sessions daily for 3 days; Meng et al. (63, 64) used two 20-min sessions daily for 5 days; Lin et al. (62) used one 40-min session daily for 10 days; and Chen et al. (58) administered acupuncture for 14 days. No study explicitly adhered to STRICTA guidelines (51). All used body acupuncture; none used scalp, ear, or laser acupuncture. One study by Liu et al. (60) used TEAS.

### 3.4 Acupoint selection

Acupoint combinations varied, but several points were used frequently. ST36 (Zusanli) appeared in 14 of 17 studies (82.3%), ST25 (Tianshu) in 6 studies (35.2%), and ST37 (Shangjuxu) in 6 studies (35.2%). GV20 (Beihai) appeared in 3 studies (17.6%), while RN12 (Zhongwan) and RN4 (Guanyuan) also appeared in 3 studies each (17.6%). The acupoints RN10, RN6, SP9, PC6, ST40, GB30, GV26, EX-B2, GB34, and RN23 each appeared in 11.8% of studies.

Several studies linked specific acupoints to clinical improvements. Xiao et al. (65) reported that stimulating ST36, GB34, PC6, and RN4 significantly increased serum CD3+, CD4+, CD8+, CD4+/CD8+ ratio, and immunoglobulins G, A, and M. Meng et al. (63, 64) applied EA to ST36 and ST37, finding reduced TNF-α and IL-1β levels, decreased intra-abdominal pressure, and improved intestinal dysfunction. Wang et al. (57) reported improved lower-extremity muscle strength after needling GB30, ST32, ST36, GB39, and LR3. For GV20, Zheng et al. (50) used EA combinations including GV20 and found significantly lower plasma CRP, IL-6, and neuron-specific enolase compared to conventional treatment alone. Lin et al. (62) applied acupuncture including GV20 and reported improved Montreal Cognitive Assessment scores along with decreased plasma CRP, IL-6, and lactate. Importantly, none of the studies provided a detailed TCM rationale for acupoint selection based on syndrome differentiation.

### 3.5 Outcomes, evaluation, and safety

#### 3.5.1 Assessment scales

Nine studies used standardized scales (APACHE II, SOFA, MoCA, MRC-SS) to evaluate acupuncture’s effectiveness. Chen et al. (58) employed six scales, while Zheng et al. (59) and Wang et al. (57) used two each. Multiple scales facilitate comprehensive evaluation, especially when acupuncture is applied to both groups. However, scale-based data should be combined with objective biomarkers to confirm efficacy (32, 75).

#### 3.5.2 Biomarkers and objective measures

Nine studies investigated short-term effects using hematological and biochemical indices; six of these also measured plasma biomarkers. Examples include: Chen et al. (58) measuring plasma MSTN and ultrasound-measured rectus femoris cross-sectional area (RFCSA); Li et al. (55) assessing WBC, hs-CRP, and PCT; Meng et al. (64) measuring PCT, TNF-α, I-FABP, D-lactate, and citrulline; Yang et al. (49) measuring CD3+, CD4+, CD8+, and CD4+/CD8+; Wang et al. (57) using ultrasound to measure quadriceps thickness and gastrocnemius plume angles; and Liu et al. (60) using ultrasound to assess gastric antral contractions. Compared to other disease areas (75), sepsis acupuncture trials report a higher proportion of matched clinical testing data, likely due to the intensive monitoring inherent to ICU care.

#### 3.5.3 Timing of assessments

All studies used pre- and post-acupuncture data. Five relied on a single pre/post comparison, while seven collected data at multiple time points. For example, Chen et al. (58) collected data on days 1, 3, 5, 7, 10, and 14; Li et al. (55) on days 1, 3, 6, and 10; and Yang et al. (49) on days 3 and 7 post-acupuncture. However, all studies focused exclusively on short-term outcomes. Only Chen et al. (58) incorporated a 1-year follow-up, but those results remain unpublished.

#### 3.5.4 Adverse events

No serious adverse events were reported. Minor events included mild restlessness, discomfort, pain, subcutaneous hematoma, and bleeding. Wang et al. (57) reported one case of mild restlessness/discomfort resolved by reducing electrical stimulation intensity. These minor events likely relate to acupuncturist technique, patient posture, or local anatomy (76). Nevertheless, given the small sample sizes and multiple influencing factors, larger cohort studies are needed to definitively establish safety.

## 4 Discussion

### 4.1 Methodological limitations of existing trials

All 17 studies reported random assignment, and most described single- or double-blinding; however, small-sample trials typically used simple randomization, which limits balance between groups. Only one study i.e., Xiao et al. (65) included an active non-acupuncture comparator (thymosin-α1), and none incorporated a SA control using placebo needles, superficial needling, or non-acupoint stimulation. This omission is significant because SA itself can elicit physiological responses similar to genuine acupuncture (77–79); for example, one study on xerostomia found that SA significantly improved saliva secretion compared to control (80). Without SA groups, the specific effects of acupuncture cannot be distinguished from placebo.

None of the 17 trials reported a priori sample size calculations based on effect-size analysis (56). Sample sizes ranged from 30 to 120 (median 60), with only one trial enrolling more than 100 participants. Inclusion and exclusion criteria were not uniformly strict; most studies did not adopt stringent criteria for specific organ dysfunctions such as SAE, ICUAW, or gastrointestinal failure (49, 58–60).

Furthermore, no trial consistently handled age as a covariate or stratification factor. This is a concern because age is routinely used to assess disease severity and quantify risk in sepsis (81–83). Nevertheless, the prognostic value of age alone remains debated: some studies report no significant differences in 1-year survival between patients aged ≥ 65 years and those aged > 85 years after adjusting for comorbidity and frailty (84–86), and clinicians accordingly hold divergent opinions on whether age independently predicts ICU mortality (81, 82, 87). The absence of age-adjusted analyses therefore leaves unresolved whether acupuncture effects might be confounded by age-related differences in baseline prognosis.

Collectively, these methodological shortcomings limit the ability to draw definitive conclusions about acupuncture’s additive benefits in sepsis.

### 4.2 Challenges in group design and control conditions

All included studies had control groups, which help correct for background differences. However, Lin et al. (62) used acupuncture in both experimental and control groups (different acupoint combinations), which is useful for studying acupoint specificity but cannot prove additional benefit over standard care alone. The absence of a no-acupuncture control group in that design limits interpretation.

Researchers have consistently reported divergent views on acupuncture’s potential placebo effects. To address this, many clinical trials in other fields incorporate SA groups to explore acupoint specificity by comparing true versus sham acupuncture (88, 89). None of the sepsis trials did so. Furthermore, the evaluation of CAM therapy outcomes often involves subjective assessments by doctors, patients, and families. Acupuncture is characterized by significant inter-practitioner variation in TCM knowledge and acupoint selection, with no recognized standards. The potential impact of subjective factors such as manual manipulation level, patients’ psychological status, and physician-patient relationship are key issues that require consideration in group design (90).

### 4.3 Intervention heterogeneity and co-intervention confounding

All experimental groups received acupuncture alongside standard pharmacotherapies (antibiotics, vasopressors) (15–17, 71, 72). Under this concurrent administration, any observed improvement could theoretically reflect the drug, acupuncture, or an interaction between them. For example, linezolid is typically given for 7–10 days and ceftazidime-avibactam require continuous or every-8-h dosing (71, 72); thus, the drug treatment window overlaps entirely with the acupuncture period. One approach to address this in future studies would be staggered initiation or factorial designs.

None of the 17 trials reported whether patients received other CAM therapies such as probiotics or herbal remedies (19, 21, 22, 73, 74). This absence of reporting is notable because previous work indicates that up to 50% of patients with psychiatric symptoms (common in SAE) use CAM (75). Accordingly, future studies could either exclude such therapies or document them systematically. Among the 17 trials, 7 used EA and 10 used MA; however, no study directly compared the two modalities, and their relative efficacy therefore remains unclear. Treatment regimens varied considerably, ranging from 3 to 14 days and involving sessions of 20–60 min. This variability makes dose-response relationships difficult to infer. None of the trials declared conformance with STRICTA guidelines (51), a factor that may limit reproducibility and cross-study comparability.

All trials used body acupuncture exclusively; scalp, ear, and laser acupuncture were not tested, despite evidence supporting their use in other conditions (91, 92). Laser acupuncture offers potential advantages such as painlessness and non-invasiveness (93), and one trial used TEAS (60) to avoid skin puncture. Whether these alternative modalities are more suitable for the ICU setting remains to be determined through comparative studies. As reported in Results section “3.5.4 Adverse events,” no serious adverse events occurred; nevertheless, given the small aggregate sample size, the safety profile should be confirmed in larger cohorts.

### 4.4 Acupoint selection: patterns, rationale, and mechanistic links

Although acupoint combinations varied across the reviewed studies, several acupoints, particularly ST36, ST25, ST37, GV20, RN12, and RN4, were used with notable consistency. However, none of the studies provided a detailed TCM rationale for acupoint selection based on syndrome differentiation, which limits readers’ understanding and may lead to one-sided mechanistic interpretations. The use of fixed acupoint combinations across trials reflects the TCM principle of “same treatment for the same syndrome with different diseases” (异病同治) (94). Whether this approach adequately addresses the diverse clinical manifestations of sepsis-related multiorgan dysfunction remains uncertain.

ST36 (*Zusanli*) – Belongs to the Stomach Meridian of *Foot-Yangming*, located between the anterior tibial and extensor digitorum longus muscles, surrounded by the lateral sural cutaneous nerve, deep peroneal nerve branches, and tibial vessels (95). In TCM theory, ST36 regulates the spleen and stomach and enhances immunity (96). Mechanistically, acupuncture at ST36 drives the vagal-adrenal axis, involving dopamine secretion and PROKR2-Cre distribution (97, 98). EA at ST36 has been shown to reduce serum TNF, IL-6, nitrite, and HMGB1 in sepsis patients, thereby improving survival (99). It also improves visceral function in irritable bowel syndrome by regulating brainstem and spinal cord serotonin neurons (100). In the reviewed studies: Xiao et al. (65) (using ST36, GB34, PC6, RN4) reported increased CD3+, CD4+, CD8+, CD4+/CD8+ ratios, IgG, IgA, and IgM; Meng et al. (63, 64) (using ST36 and ST37) reduced TNF-α, IL-1β, and IAP while improving intestinal dysfunction; Wang et al. (57) (using GB30, ST32, ST36, GB39, LR3) improved lower-extremity muscle strength.

GV20 (*Baihui*) – Located on the head, GV20 belongs to the *Governor Vessel* (督脉) and is surrounded by the galea aponeurotica, greater occipital nerve, and superficial temporal and occipital vessels. It intersects the *Governor Vessel* with the Hand and Foot Three *Yang Meridians*, making it suitable for various diseases (48), including dementia, stroke, insomnia, epilepsy, hypertension, and schizophrenia (48, 101–103). Given that up to 70% of sepsis patients develop sepsis-associated SAE, this explains GV20’s frequent use in sepsis studies (104, 105). Potential mechanisms include increased dopamine secretion and restored hippocampal long-term potentiation to improve memory (106, 107). EA at GV20 and GV14 has been shown to reduce apoptosis, oxidative stress, and inflammation while improving cerebral blood perfusion in ischemic stroke mice (108). Stimulation of GV26, GV20, and HT7 activates glucose metabolism in the frontal lobes, thalamus, temporal lobe, and putamen, thereby improving vascular dementia (109, 110). In the reviewed studies by; Zheng et al. (59) (EA including GV20) found lower plasma CRP, IL-6, and NSE; Lin et al. (60) (acupuncture including GV20) improved MoCA scores and decreased CRP, IL-6, and lactate. However, Lin’s group setting (acupuncture administered in both groups) cannot determine whether acupuncture provides additional benefit over conventional treatment alone.

ST37 (Shangjuxu) – Located on the anterolateral aspect of the lower leg, one finger’s breadth (index finger) from the anterior margin of the tibia, ST37 belongs to the *Foot-Yangming* Stomach Meridian and serves as a lower He-Sea point for the large intestine. According to traditional TCM theory, it has the effects of regulating the digestive system, clearing heat from the intestines, and supporting the body’s constitution. In this review, Meng et al. (64) used EA at ST37 as an adjunct to conventional treatment. The results showed that it significantly reduced levels of intestinal fatty acid binding protein (I-FABP) in sepsis patients, indicating reduced intestinal mucosal damage. Additionally, serum levels of TNF-α and IL-1β exhibited a downward trend, suggesting reduced inflammatory responses. Liu et al. (60) found that EA at ST37 combined with conventional Western medical treatment significantly promoted gastrointestinal motility function and improved early tolerance to enteral nutrition in sepsis patients, thereby shortening the time required to achieve enteral nutrition standards and reducing hospital stay. The efficacy was superior to that of conventional Western medical treatment alone.

ST25 (*Tianshu*) – An abdominal acupoint, ST25 is a critical point on the *Foot-Yangming* Stomach Meridian and the *Mu-Front* point of the large intestine. It possesses unique advantages in directly affecting the intestines, regulating *qi*, and resolving stagnation, making it one of the key acupoints in clinical research. Studies have shown that ST25 may exert a positive regulatory effect on intestinal barrier damage and systemic inflammatory state associated with sepsis by modulating both local and systemic immune responses (44, 46, 47). In this review, Wu et al. (66) demonstrated that acupuncture at ST25, applied in addition to conventional treatment, improves intestinal permeability in septic patients, facilitating rapid restoration of intestinal function and early achievement of targeted feeding. Sun et al. (67) and Wei et al. (69) found that acupuncture at ST25 improved inflammatory markers, APACHE II scores, intra-abdominal pressure, gastric residual volume, and bowel sounds in patients with sepsis.

In summary, the use of acupoint combinations aligns with TCM’s holistic approach but complicates the evaluation of individual acupoint efficacy. Nevertheless, comparative analysis of the most frequently used acupoints can inform future “gold standard” development.

### 4.5 Outcome measures: scales, biomarkers, and follow-up

The use of standardized scales (APACHE II, SOFA, MoCA, MRC-SS) across nine trials reflects appropriate attention to clinically meaningful endpoints. However, as noted in Results, scale-based data were not consistently combined with objective biomarkers, a missed opportunity for cross-validation (32, 75). Notably, sepsis acupuncture trials reported a higher proportion of matched clinical testing data than studies in other disease areas (75), likely reflecting the intensive monitoring inherent to ICU care.

Nevertheless, all objective measures were limited to short-term outcomes (days to weeks), leaving the durability of acupuncture’s effects unaddressed. Long-term follow-up is essential, as other conditions have demonstrated benefits persisting for 3–6 months (111, 112). Yet, the only sepsis trial that included a 1-year follow-up has not published its results (51), leaving this critical gap unresolved.

### 4.6 Adverse events: interpretation

The absence of serious adverse events across all 17 trials suggests acupuncture is well-tolerated in critically ill sepsis patients, consistent with broader acupuncture safety literature (76). However, several limitations temper this conclusion. First, minor event reporting may be incomplete due to the complexity of ICU monitoring. Second, small sample sizes (median 60) preclude detection of rare events. Third, the lack of standardized safety reporting across trials, none followed STRICTA guidelines for adverse events, hinders meta-analysis. Finally, generalizability to real-world populations remains uncertain, as trial patients may differ from clinical populations in hemodynamic stability, coagulation status, and concurrent treatments. Future trials should adopt standardized safety reporting frameworks to enable meaningful comparisons.

### 4.7 Future prospects

Based on our analysis, acupuncture appears to be a feasible complementary therapy for sepsis. However, the current evidence is limited by small sample sizes and methodological shortcomings, preventing its unanimous endorsement. Therefore, to strengthen the evidence base, future research must adopt more scientifically rigorous designs. This includes adhering to STRICTA and CONSORT reporting guidelines, using the Oxford Center for Evidence-Based Medicine Level of Evidence Scale, and accounting for key confounding factors (52, 54, 73). From the perspective of TCM theory, the treatment emphasizes different conditions of each patient, which is based on syndrome differentiation. Therefore, in addition to applying strict inclusion criteria, the different syndromes of sepsis should also be considered in study design. Furthermore, protocols must be developed to objectively evaluate the specific contribution of acupuncture alongside concurrent modern medical therapies.

As shown in Table 1, acupoint selection differed substantially among the trials. While all studies asserted their combinations were suitable for sepsis, the lack of comparative data precludes identifying an optimal protocol. In the absence of a “gold standard,” repeated multicenter trials with large cohorts are needed to screen and validate the most effective acupoints and combinations. Furthermore, the experimental groups in these trials should be established with the goal of screening the best applicable acupoints, and multiple acupoint combinations should be tested using factorial designs that systematically pair candidate “main” acupoints with “matching” acupoints (76). Similarly, given the need for more objective evidence to support the efficacy of acupuncture, the study results should be supplemented with substantial clinical testing data obtained using scales to address aspects such as sepsis-related inflammation, immune, coagulation, pathogenic microorganisms, and toxins (56, 113). In addition, regular follow-up assessments of the study participants can further clarify the duration of acupuncture effects, thereby expanding the evidence base (83, 113).

Traditional Chinese medicine and modern medicine are distinct theoretical systems. Bridging this divide to elucidate the mechanisms of acupuncture in sepsis is a significant challenge, complicated by the complexity of meridian theory, the diversity of acupoint selection, and the incomplete understanding of sepsis pathogenesis (7, 114). However, research on emerging systems biology approaches is beginning to address this. Techniques such as imaging, multi-omics, network pharmacology, and bioinformatics are being used to investigate the biological basis of TCM syndromes. A growing body of research on meridian visualization also contributes to this effort (115–117). Together, these advancements provide a valuable foundation for exploring the potential mechanisms of acupuncture in sepsis.

### 4.8 Limitations

This review has several limitations. First, the narrative synthesis precludes meta-analysis. Second, restricting to English and Chinese publications may introduce language bias. Third, published studies only risk publication bias. Finally, the small number of eligible trials and their heterogeneity limit generalizability.

## 5 Conclusion

Current clinical trials do not provide robust evidence for the additive benefits of acupuncture in sepsis. Nevertheless, they form a critical foundation for future research. To build upon this groundwork, we recommend strict adherence to STRICTA, CONSORT, and Oxford Center for Evidence-Based Medicine guidelines. Future studies should prioritize identifying optimal acupoint combinations and elucidating individual acupoint functions. This review synthesizes existing evidence and offers design-level recommendations to bridge TCM theory and modern science. Further investigation is needed to understand interactions between conventional medical therapies and concurrently or sequentially administered acupuncture.
